# Gut Microbiota as Diagnostic Tools for Mirroring Disease Progression and Circulating Nephrotoxin Levels in Chronic Kidney Disease: Discovery and Validation Study

**DOI:** 10.7150/ijbs.37421

**Published:** 2020-01-01

**Authors:** I-Wen Wu, Chan-Yu Lin, Lun-Ching Chang, Chin-Chan Lee, Chih-Yung Chiu, Heng-Jung Hsu, Chiao-Yin Sun, Yuen-Chan Chen, Yu-Lun Kuo, Chi-Wei Yang, Sheng-Siang Gao, Wen-Ping Hsieh, Wen-Hung Chung, Hsin-Chih Lai, Shih-Chi Su

**Affiliations:** 1 Department of Nephrology, Chang Gung Memorial Hospital, Keelung, Taiwan; 2 College of Medicine, Chang Gung University, Taoyuan, Taiwan; 3 Kidney Research Center, Department of Nephrology, Chang Gung Memorial Hospital, Linkuo, Taiwan; 4 Department of Mathematical Sciences, Florida Atlantic University, Florida, US; 5 Department of Pediatrics, Chang Gung Memorial Hospital, Keelung, Taiwan; 6 Biotools, Co., Ltd, New Taipei City, Taiwan; 7 Institute of Statistics, National Tsing-Hua University, Hsinchu, Taiwan; 8 Whole-Genome Research Core Laboratory of Human Diseases, Chang Gung Memorial Hospital, Keelung, Taiwan; 9 Graduate Institute of Biomedical Sciences, Division of Biotechnology, College of Medicine, Chang Gung University, Taoyuan, Taiwan; 10 Microbiota Research Center, Chang Gung University, Taoyuan, Taiwan.

**Keywords:** Chronic kidney disease, gut microbiome, p-cresyl sulfate, and indoxyl sulfate

## Abstract

The interplay of the gut microbes with gut-producing nephrotoxins and the renal progression remains unclear in large human cohort. Significant compositional and functional differences in the intestinal microbiota (by 16S rRNA gene sequencing) were noted among 30 controls and 92 (31 mild, 30 moderate and 31 advanced) patients at different chronic kidney disease (CKD) stages (discovery cohort). A core CKD-associated microbiota consisted of 7 genera (Escherichia_Shigella, Dialister, Lachnospiraceae_ND3007_group, Pseudobutyrivibrio, Roseburia, Paraprevotella and Ruminiclostridium) and 2 species (Collinsella stercoris and Bacteroides eggerthii) were identified to be highly correlated with the stages of CKD. Paraprevotella, Pseudobutyrivibrio and Collinsella stercoris were superior in discriminating CKD from the controls than the use of urine protein/creatinine ratio, even at early-stage of disease. The performance was further confirmed in a validation cohort comprising 22 controls and 76 peritoneal dialysis patients. Bacterial genera highly correlated with indoxyl sulfate and p-cresyl sulfate levels were identified. Prediction of the functional capabilities of microbial communities showed that microbial genes related to the metabolism of aromatic amino acids (phenylalanine, tyrosine, and tryptophan) were differentially enriched among the control and different CKD stages. Collectively, our results provide solid human evidence of the impact of gut-metabolite-kidney axis on the severity of chronic kidney disease and highlight a usefulness of specific gut microorganisms as possible disease differentiate marker of this global health burden.

## Introduction

Chronic kidney disease (CKD), which has striking comorbidities with metabolic and cardiovascular disease and could progress into end-stage renal disease (ESRD), is a serious public health dilemma. According to the registries of different countries, CKD affects 8%-16% of adults around the world 1. Within the Taiwanese population, the estimated prevalence of CKD is 11.9% 2, as both the incidence and prevalence of ESRD in Taiwan are among the highest in the world 3. In addition to genetic components, major causes of CKD include but not limited to age, obesity, hypertension, and diabetes mellitus 4. Identification of predisposing factors to CKD is essential for the development of preventive and therapeutic strategies against this global health issue, as some risk factors can be manipulated, thereby impeding the disease progression to ESRD.

The intricate interaction between the human gut microbiota and the host is central to the development and progression of many human diseases 5. In spite of considerable variations between intestinal microbiota in rodents and in humans 6, mounting evidence has revealed profound alterations of gut microbiota in patients and animals with CKD 7, 8. For human gut flora, although inconclusive, compositional differences, with a profusion of *Firmicutes*, *Actinobacteria*, and *Proteobacteria*, and a significant reduction in the abundance of *Bifidobacteria* and *Lactobacilli*, were detected in patients with ESRD as compared to normal controls 9-11. Of note, the intestinal microbiota can be highly adaptable to fluctuations in the biochemical milieu at the early stage of this syndrome, as relevant quantitative and qualitative changes in the microbial population of CKD patients have been demonstrated 12. Such dysbiosis of gut microorganisms, together with impairment of the intestinal epithelial barrier function are known to contribute to the pathogenesis of systemic inflammation in CKD by accommodating the translocation of endotoxin, microbial fragments and other noxious luminal products in the circulation 13.

In addition to regulation of the biochemical microenvironment of the gastrointestinal tract and host immunity, gut bacteria convert diet-derived molecules into hundreds of diffusible organic compounds, some of which are toxic and must be cleared from the body. Many of these molecules are normally excreted in urine. In patients with CKD, however, kidney function remains diminished for a long period, and these compounds accumulate in serum and contribute to disease progression 14-16. Two such compounds, p-cresyl sulfate (pCS) and indoxyl sulfate (IS), in CKD patients can lead to renal and cardiovascular damage 17. In an animal model of CKD, *Anaerotruncus colihominis*, *Clostridium citroniae*, *Clostridium saccharolyticum*, and *Paludibacter propionicigenes* were identified as the candidate IS and pCS-producing microbiota in the rat intestine 18. Two bacterial operational taxonomic units (OTUs), belonging to the genus *Oscillospira* and the family *Ruminococcaceae*, were reported to be associated with urinary levels of pCS in healthy subjects 19. Similarly, an elevated level of circulating pCS was found to be correlated with the increased abundance of the *Ruminococcus* genus in the feces of patients with early-stage CKD 12. To date, studies of gut microbiome on CKD have been focused on either animal model or patients having advanced stages of disease 9-11, 20-22 or early renal function decline 12. Yet, the exact correlation of the gut microorganisms with gut-producing nephrotoxins and the disease progression in CKD patients having different disease stages remains largely unclear. Hence, in the present study, we aim to explore the altered intestinal microbiota in patients with different stages of CKD and their relationship with serum levels of bacteria-derived uremic toxins.

## Materials and Methods

### Subjects

100 patients with CKD and 30 subjects with normal renal function and matched age, gender, and status of diabetes and hypertension were recruited in the Department of Nephrology, Chang Gung Memorial Hospital, Keelung, Taiwan. CKD was defined as either the presence of proteinuria or an estimated glomerular filtration rate (eGFR), determined by using simplified Modification of Diet in Renal Disease equation, of less than 60 ml/min/1.73 m2 in two separate occasions and classified into stage 1 to 5, according to the NKF/DOQI classification 23. For exploration of the disease progression, patients were divided into mild (stage 1 and 2, n=31), moderate (stage 3, n=30) and advanced (stage 4 and 5, n=31) CKD. In addition, a minor group of 8 advanced patients treated with AST-120 (Kremezin 6g/day for 12 weeks) was used as an additional control, and a replication cohort, including 22 controls and 76 stable peritoneal dialysis (PD) patients, was enrolled from Chang Gung Memorial Hospital at Linkuo for verification of clinical validity. Based on effect size of 40% and significance level at 0.05 under two-tail analysis, a minimal of 112 total samples was found to have a study power of 0.95 and ⍺-error probability of 0.05 in a 4-groups design (non-CKD, mild, moderate and advanced CKD groups). A study number of 122 patients (at least 30 per group) were justified by sample size calculation statement. Similarly, based on effect size of 40%, β/⍺ ratio of 0.05, the sample of 22 vs.78 patients, was found to have a study power of 0.95 by using non-parametric design for health and PD groups in the validation cohort. All participants provided informed written consent at enrollment. Patients with malignancy, liver cirrhosis, intestinal operation, irritable bowel syndrome, cardiovascular disease (defined as myocardial infarction, documented Q wave on electrocardiogram, unstable angina, coronary artery disease with stenosis >75%, congestive heart failure with an ejection fraction <50% and cerebrovascular disease), active infection, concomitant use of probiotics, prebiotics or antibiotics, pregnancy or renal transplant recipients were excluded from study. All patients had regular 3-meals dietary pattern. Vegetarian or people on vegan diets were also excluded to avoid distortion on the dietary pattern of entire cohort. This study was conducted in adherence to the Declaration of Helsinki and approved by the Institutional Review Board at Chang Gung Memorial Hospital (IRB-102-5507A3 and 104-1145C). The informed consent was obtained from all patients.

### Measurement of gut-producing metabolites

Circulating pCS and IS (free and protein-bound fractions) were analyzed with ultraperformance liquid chromatography-tandem mass spectrometry (UPLC-MS/MS, Milford, MA, USA) in all patients of discovery cohort. Concentrations of free pCS and IS were measured in serum ultrafiltrates by using AmicoUltra 30 K filter (Millipore). 100 μL of samples were deproteinized by addition of 4 parts of acetonitrile. Chromatographic separation was performed at 30°C using a Acquity UPLC BEHC 18 column (2.1 x 100mm). The separation was run for a total of 5.5 minutes using a gradient elution composed of solvent A (0.1% Formic acid) and solvent B (1mM NH4OAc +0.1% formic acid in 100% acetonitrile). The analytes were quantified with a Waters Acquity UPLC Xevo TQ-S operating in negative electrospray ionization and multiple reaction monitoring mode 24 .

### Stool DNA isolation and 16S rRNA gene sequencing

Within 7 days before sample collection, subjects were not allowed to take any supplement or food containing probiotics such as yogurt. Bacterial DNA from stool was extracted by using the FastDNA SPIN Kit for Feces (MP Biomedical). Polymerase chain reaction (PCR) was used to amplify the variable region 4 (V4) of the gene that encodes for 16S rRNA in bacteria. The V4 region of the 16S rRNA gene was amplified by using bacteria/archaeal primer 515F/806R with the barcodes 25. Amplicons were purified by using the GeneJET Gel Extraction Kit (Thermo Scientific) and then quantified using a Qubit dsDNA HS Assay Kit (Qubit) on a Qubit 2.0 Fluorometer (Qubit). Sequencing libraries were generated using the NEB Next® Ultra™ DNA Library Prep Kit for Illumina (NEB) following manufacturer's recommendations. Purified libraries were quantified, normalized, pooled, and applied for cluster generation and sequencing on an Illumina HiSeq 2500 platform to generate 250 bp paired-end reads.

### Processing and analysis of sequence data

Paired-end reads were merged using FLASH v1.2.7 26, and quality filtering of reads was evaluated by the QIIME 1.7 pipeline using Python scrips 27. Chimeric sequences were discarded by UCHIME 28. The processed sequencing reads (effective tags) were clustered into OTU at 97% sequence identity using the UPARSE 29, and taxonomy classification was assigned according to the information retrieved from the SILVA database 30. To evaluate the phylogenetic relationship of different OTUs, alignment of multiple sequences was conducted using the PyNAST software v.1.2 31 against the dataset of the SILVA database, and a phylogenetic tree was generated with the FastTree 32. For estimating alpha diversity, species richness was evaluated by the Chao1 index at the OTU level. A rarefaction curve was generated by a random selection of certain amount of sequencing data from each sample for representing the number of the observed species, and a species accumulation curve was plotted by the occurrence rate of new OTUs (species) under continuous sampling. For evaluating beta diversity, Bray-Curtis dissimilarities at the OTU level were calculated and compared with vegan 33. Principal coordinate analysis (PCoA) was performed using the Bray-Curtis distance. Weighted and unweighted UniFrac parameters 34 were calculated by using the QIIME pipeline. Non-metric dimensional scaling (NMDS) was conducted using the weighted correlation network analysis (WGCNA), stat, and ggplot2 packages in R software by transforming a distance matrix of weighted or unweighted UniFrac parameters among samples into a new set of orthogonal axes. All pipelines and analyses are processed using in-house R scrips, unless otherwise indicated.

Functional composition of metagenomes was predicted from 16S rRNA data by the phylogenetic reconstruction of unobserved states (PICRUSt) software using Python scrips 35. A table of gene copy numbers for each gene family in each sequenced bacterial and archaeal genome based on the IMG database 36 and a phylogenetic tree from the Greengenes database 37 were precomputed for gene content prediction.

### Statistics analysis

Descriptive statistics are expressed as the mean, median or frequency. Normality of numerical variables is tested with the Kolmogorov-Simirnov method. Differences in clinical indices among groups were determined using Student's t-test or Kruskal-Wallis test. Spearman's correlation was used to determine the association of major genera (>0.1% abundance and present in >90% of samples) with serum biomarkers and disease severity, and p values were adjusted by using Bonferroni correction for multiple tests (n=55). Data were analyzed using SPSS 22.0 for Windows XP (SPSS Inc., Chicago, IL). Chao1 index was analyzed using Kruskal-Wallis test, and Bray-Curtis distance between groups was calculated by Wilcoxon rank sum test. Statistically significant biomarkers were evaluated by the linear discriminant analysis (LDA) of effect size (LEfSe) analysis, which employed the non-parametric factorial Kruskal-Wallis test, Wilcoxon rank sum test and LDA to identify differentially abundant taxa between two metadata classes. Taxa important for classifying CKD stages were identified using Random Forests 38, which ranked OTUs based on their ability to discriminate among the groups, while taking into account the complex interrelationships in high dimensional data. We included the overall taxonomic, genus-level, or species-level abundances to determine the most discriminatory taxa across different CKD statuses. The performance of gut microbial abundance for distinguishing different stages of CKD or peritoneal dialysis patients from non-CKD controls was tested by receiver operating characteristic (ROC) curves constructed by SPSS. Statistical difference between the area under curve (AUCs) was calculated by using a two-sample Z-test and adjusted with Bonferroni correction for multiple tests (n=80, for species present in >80% of samples; n=98, for genus present in >90% of samples). Difference in relative abundance of predicted microbial genes related to amino acid metabolism between groups was measured using Student's t test. All statistical tests are two-tailed, and a p < 0.05 is considered statistically significant.

## Results

### Subject characteristics

In the present study, 92 untreated (31 mild, 30 moderate and 31 advanced CKD) and 8 treated (with AST-120, as an additional control group) CKD patients were recruited. Since specific dysbiosis of gut microbiome has been linked to diabetes 39 and hypertension 40, two most common comorbidities in CKD patients, 30 non-CKD controls with normal renal function and matched age, gender, and status of diabetes mellitus and hypertension were enrolled to rule out potential confounding factors. Table 1 summarizes the baseline characteristics of discovery cohort. The mean age of validation cohort was 54.1 ± 11.0 for PD patients and 47.5 ± 8.4 years for controls, respectively. Thirty-nine (39.8%) of them were men (38.5% of PD patients and 41% of controls) and 16.7% had diabetes mellitus. The mean systolic pressure was 142.5 ± 2.2 and diastolic pressure was 82.0 ± 12.9 mmHg; body mass index, 24.4±3.8 Kg/m^2^; blood urea nitrogen, 71.9±18.1mg/dL; serum creatinine, 12.6 ± 2.6mg/dL; hemoglobin, 10.4 ± 1.5g/dL; serum albumin, 3.9 ± 0.3 mg/dL; hs-CRP, 5.9±7.9mg/L and estimated protein intake was 66.9 ± 14.2 g/day in the PD group. The waist was not measured due to abdominal distension secondary to PD fluid dwelling. The urine protein-creatinine ratio was not checked in because of anuric states in most of the PD patients.

### Microbial composition and diversity in different stages of CKD

**Figure 1:**
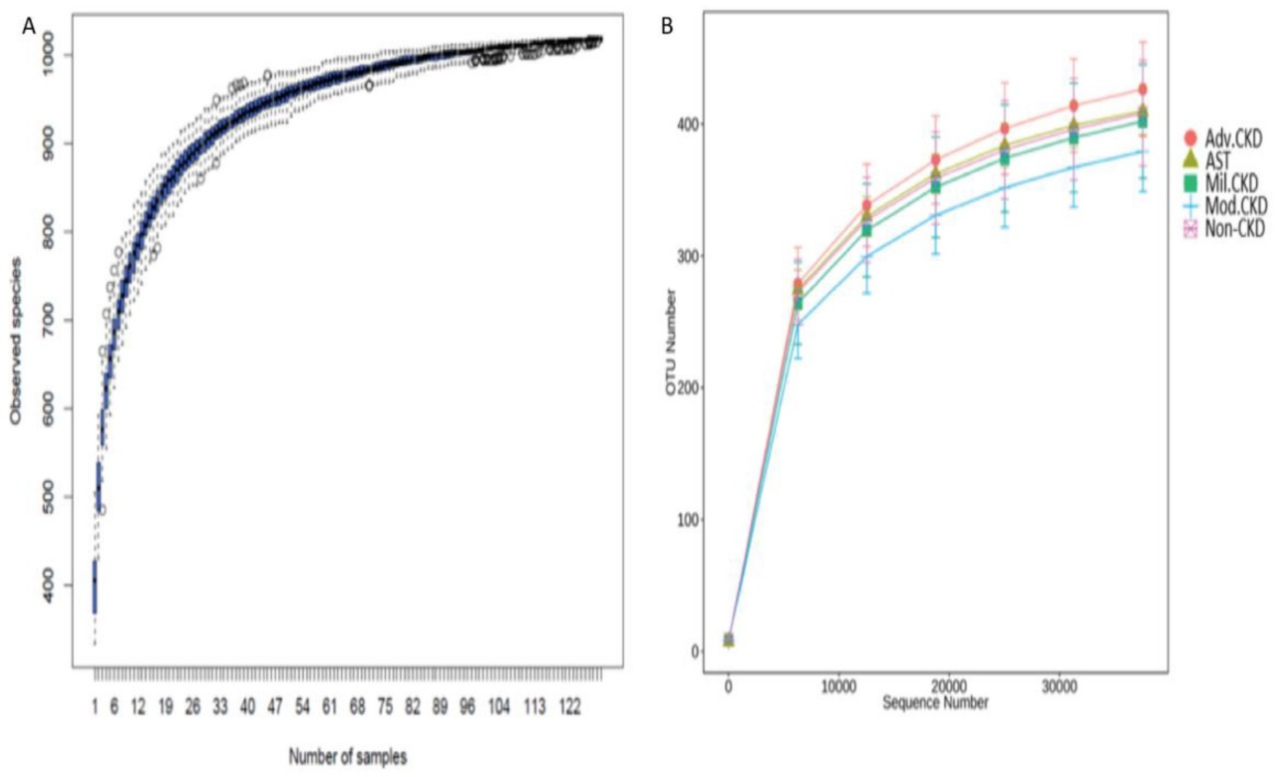
**(A)** Species accumulation curve of the gut bacterial communities detected in non-CKD controls and CKD patients. The line indicates the averaged accumulated increase of detected OTUs vs. number of samples. The box-plots show the mean, the 25th, and the 75th percentile at each sample size. **(B)** Rarefaction curves show the number of sequence reads and their corresponding number of OTUs across different groups. Mil. CKD (mild CKD); Mod. CKD (moderate CKD); Adv. CKD (advanced CKD); AST (advanced CKD with the treatment of AST-120).

A total of 7,185,764 sequencing reads, ranging from 38,134 to 66,365 per sample, was generated. After strict quality and size filtering, a total of 1024 OTUs was assigned (per sample range 333-504, Figure 1A). Rarefaction curves show that a plateau of species richness (up to 400 OTUs) was achieved in approximately 30,000 reads per sample (Figure 1B), indicating that sequencing depth we conducted covered considerable information about total species richness.

**Figure 2:**
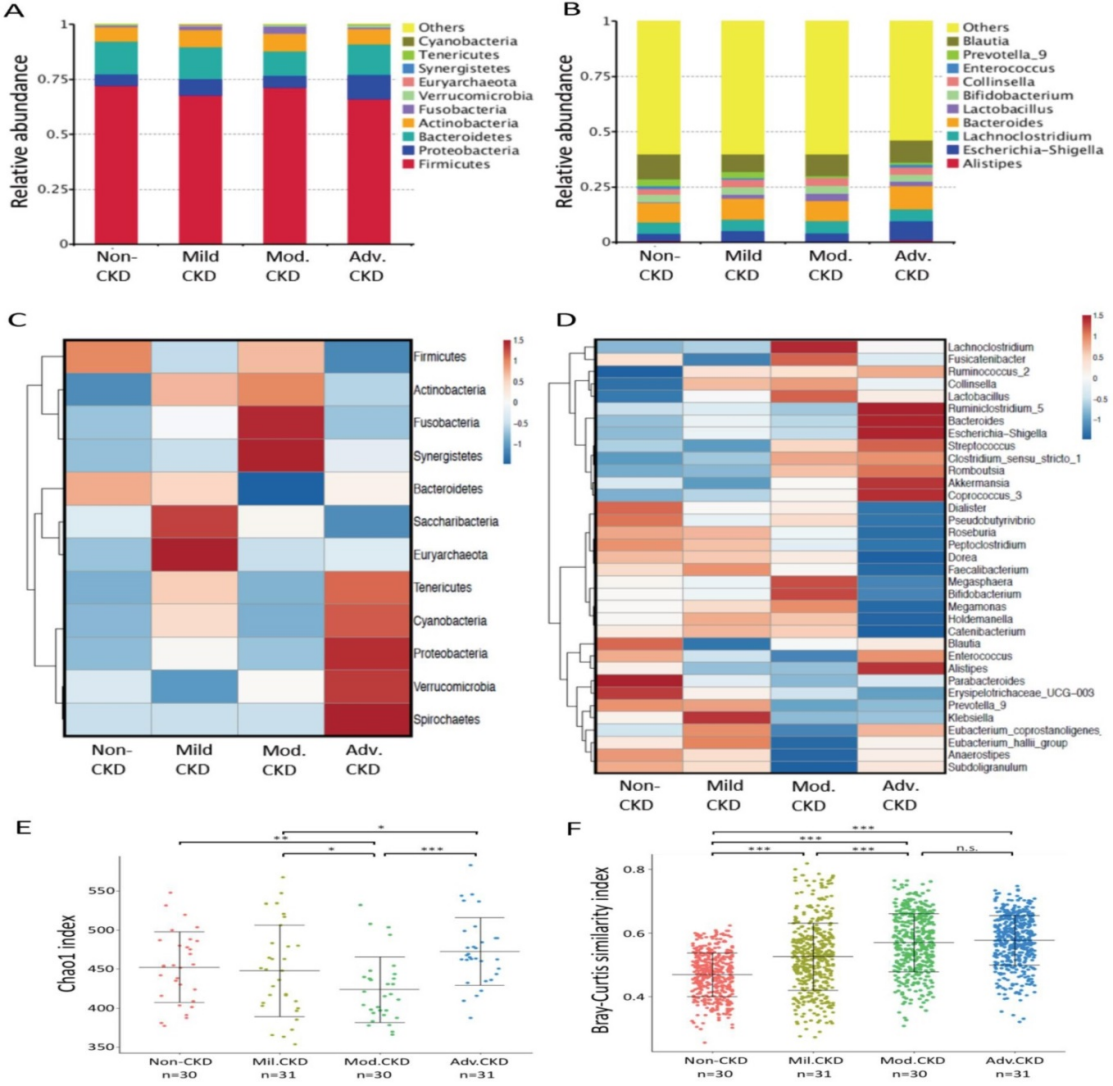
** Analysis of gut microbiota composition and diversity in discovery cohort by CKD stages. (A)** The distribution of top 10 phyla and top 10 genera **(B)** detected in different phenotypic subgroups. Heatmaps of top 12 phyla **(C)** and top 35 genera **(D)** associated with different groups are shown. **E)** α-diversity (Chao 1) and **(F)** β-diversity (Bray-Curtis similarity index) of gut microbial communities in different CKD stages. The box-plot shows the median, the 25th, and the 75th percentile in each group. Chao1 index was analyzed using Kruskal-Wallis test, and Bray-Curtis distance between groups was calculated by Wilcoxon rank sum test. *,* p* <0.05; **,* p* <0.01; ***,* p* <0.001; n.s., not significant. Mil. CKD (mild CKD); Mod. CKD (moderate CKD); Adv. CKD (advanced CKD).

Taxonomic analysis identified* Firmicute*, *Proteobacterias*, *Bacterioidetes*, and *Acinobacterias* as the most predominant taxa at the phylum level among different stages of CKD patients (Figure 2A). At lower taxonomic level, the most abundant genera were *Bacteroides*, *Blautia*,* Escherichia-Shigella*, *Collinsella*, *Lachnoclostridium*, and *Lactobacillus* in CKD patients (Figure 2B). Analysis of bacterial composition and abundance categorized by disease severity revealed that the relative abundance of many taxa at the phylum and genus levels differed among non-CKD controls and different CKD groups (Figure 2C, 2D). Significant differences in bacterial species richness and evenness (alpha diversity) were detected among different CKD stages (Figure 2E), indicating variations in gut microbiome throughout the course of CKD. Analyses of sample-to-sample dissimilarities in bacterial community structures (beta diversity) demonstrated a notable discrimination among different stages of CKD and non-CKD controls (Figure 2F), suggesting a usefulness of gut microbiome in monitoring prognosis of this disease. Notably, we observed an increase in the heterogeneity of community structure with the disease severity. Yet, this increase halted in moderate CKD as no significant difference in sample-to-sample dissimilarities was detected between moderate and advanced CKD.

**Figure 3:**
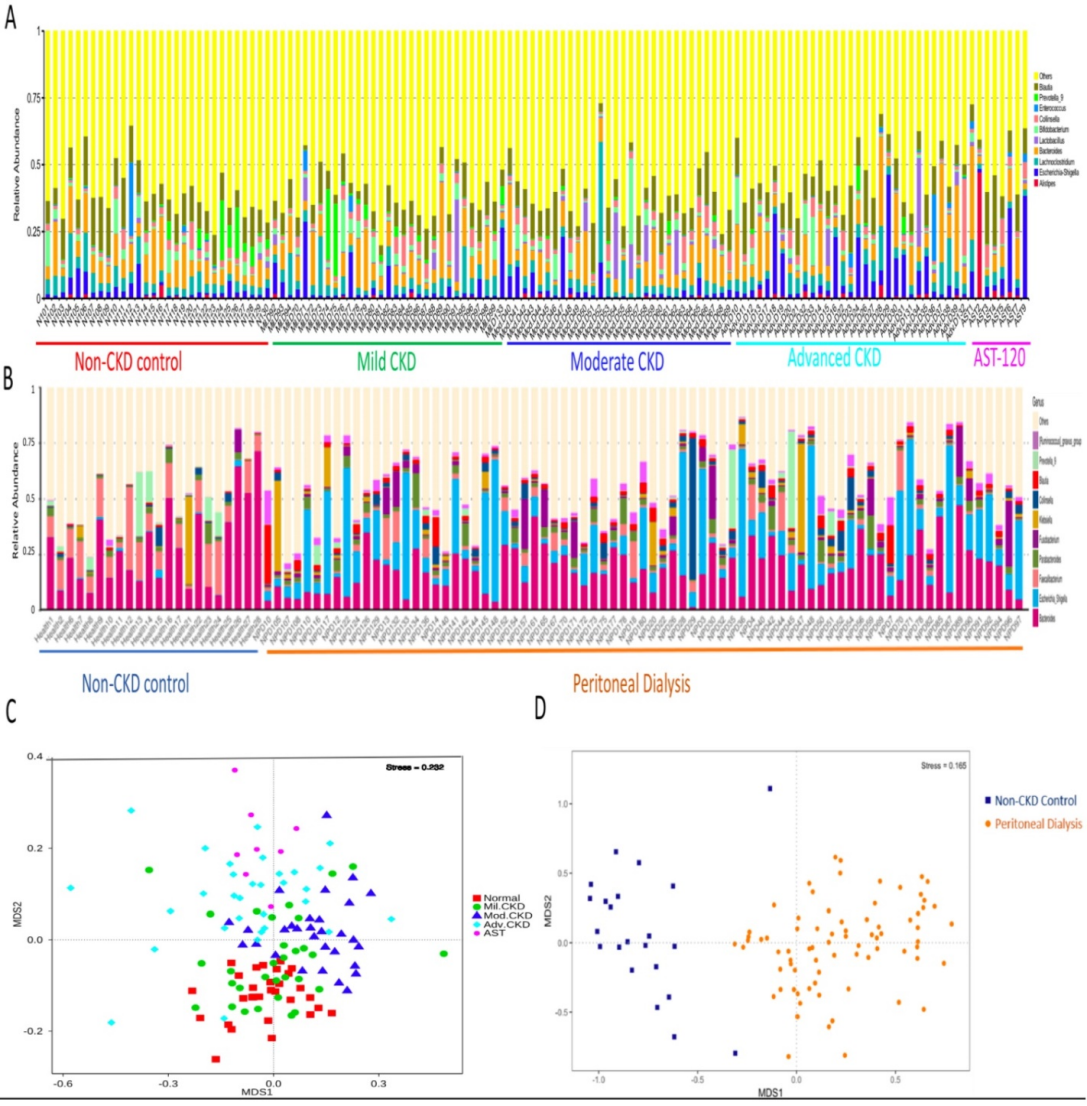
**Analysis of gut microbiota composition and diversity in discovery and validation cohort.** Distribution of top 10 genera among fecal samples of participants of discovery **(A)** and validation cohorts **(B).** Nonmetric multidimensional scaling (NMDS) ordination displaying gut microbial communities of different stages of CKD **(C)** and between non-CKD controls and PD patients **(D)**.

The difference on gut microbial composition and diversity was further validated in PD and non-CKD patients, suggesting a usefulness of gut microbiome in monitoring severity of CKD (Figure 3).

### Bacterial biomarker correlated with different stages of CKD

**Figure 4:**
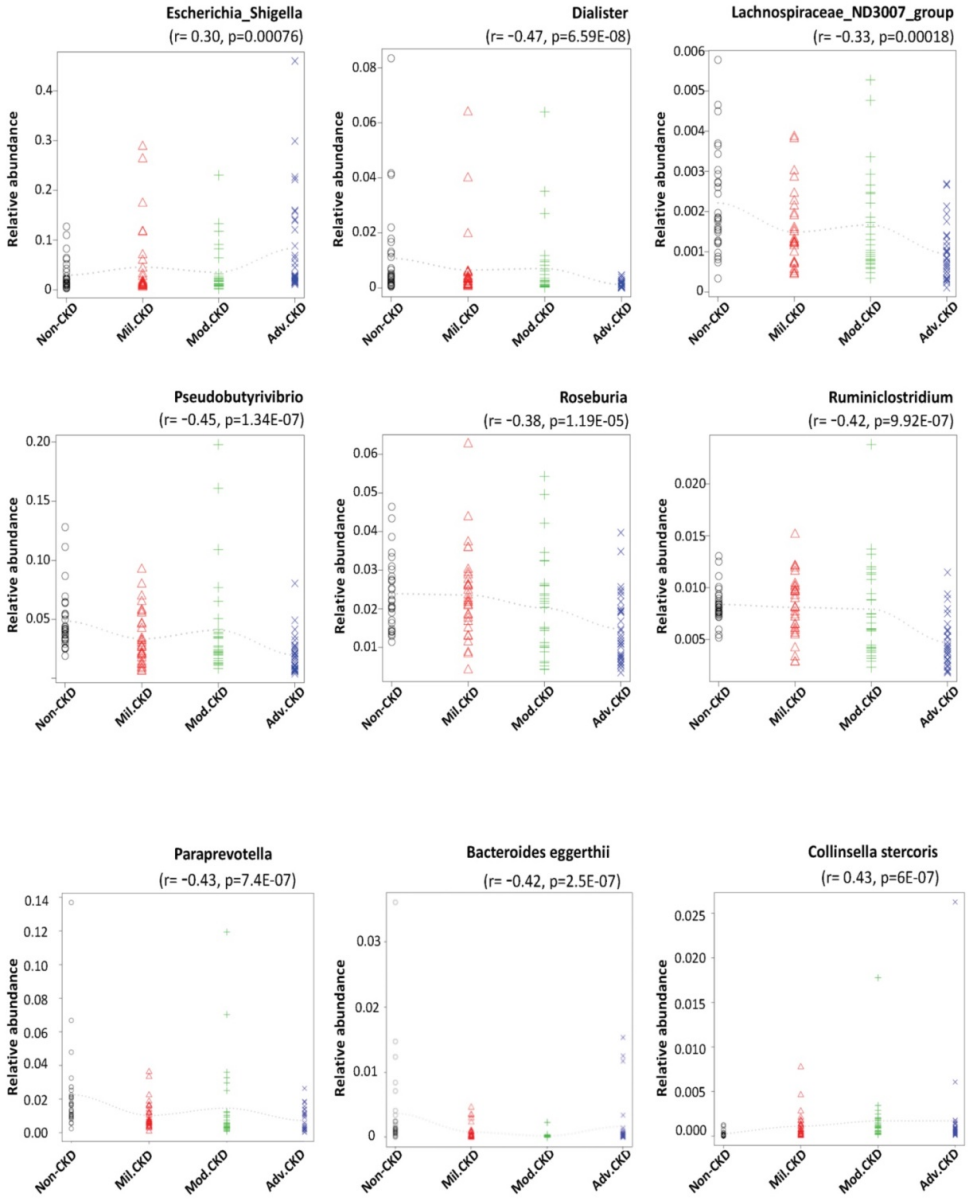
**Core CKD-associated microbiota significantly correlated with different disease stage.** Spearman's correlation indexes (r) and p values (p) are shown under the genus of bacteria. The locally weighted scatterplot smoothing (LOWESS) regression curves indicate the trends of correlations between the levels of bacterial genera and the progression of CKD. Mil. CKD (mild CKD); Mod. CKD (moderate CKD); Adv. CKD (advanced CKD).

Using rigorous criteria (>0.1% abundance and present in >90% of samples), 7 major genera and 2 species, as the core CKD-associated microbiota, were identified to be highly correlated with the various stages of CKD (Figure 4). We found that levels of *Escherichia_Shigella* spp. were positively correlated with the disease course, while that of *Dialister*, *Lachnospiraceae_ND3007_group*, *Pseudobutyrivibrio*, *Roseburia*, *Ruminiclostridium* spp**.** appeared to be negatively correlated with CKD severity.

**Figure 5:**
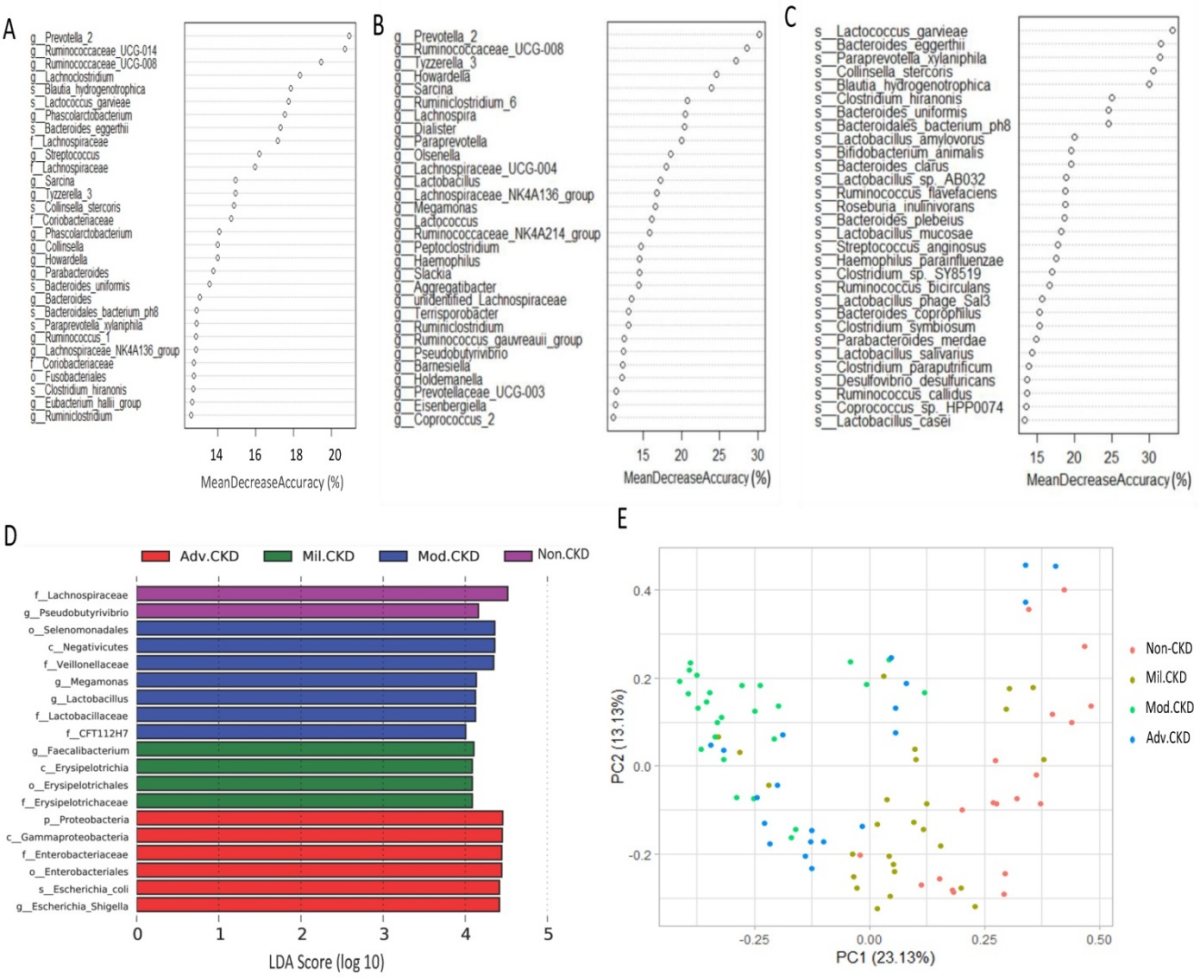
** Determination of bacterial biomarkers specific for each CKD stage or most discriminatory across different disease stages.** CKD severity-discriminatory taxa were determined by applying Random Forests analysis using the overall OUT **(A)**, only genus-levels abundance **(B)** or only species-level abundance **(C)** dataset against CKD stages. Bacterial taxa that are most discriminatory across different CKD stages were ranked in descending order of their importance to the accuracy of the model. Importance was determined based on the percentage of mean decrease in accuracy of microbiota prediction when the relative abundance of each taxon was randomly permuted. **(D)** Bacterial taxa that best characterize each CKD stage were identified by using linear discriminant analysis of effect size (LEfSe) on OTU tables. E) Principal coordinate analysis (PCoA) based on Bray-Curtis distance calculated by using top 10 discriminatory taxa identified in (A) among samples of different groups. The percentage in each axis indicates how much variation explained.

Moreover, extensive analyses were performed to unveil microbial taxa that mirror the progression of CKD. We applied the Random Forests model using the overall (1024 OTUs, Figure 5A), only the genus- (246 OTUs, Figure 5B), or the species-level (180 OTUs, Figure 5C) microbiota profiles to determine the most discriminatory taxa. Consistently, analyses of three datasets revealed the top discriminatory taxa across different disease stages. Approximately 85% of samples can be correctly classified based on the abundance of 1024 OTUs detected, while roughly 75% can be predicted if using solely the genus- or species-level abundance (Figure 5A, 5B, 5C). Furthermore, we predicted the biomarker for each stage and the non-CKD group by taking statistical significance and biological consistency into consideration using LEfSe. Among these potential biomarkers, samples in the advanced CKD group had significant enrichment for genus *Escherichia_Shigella* as compared with those in the other groups. On the contrary, a profusion of genus *Pseudobutyrivibrio* was observed in the non-CKD controls over different stages of CKD (Figure 5D). The PCoA, calculated by using top 10 discriminatory taxa identified in Figure 5B, demonstrated clear separation of moderate and advanced CKD from non-disease controls (Figure 5E), suggesting the predictive value of a selected panel of gut microbes for classification of CKD patients with prognostic implications.

### Clinical validity of gut microbiota in discriminating CKD

Despite different methods used above, a high degree of consistency in determining bacterial biomarkers that reflect the stages of CKD emerged from these data. We, thus, further evaluated the clinical validity of these CKD-associated intestinal microbes by constructing ROC curves for different levels of CKD severity (Table 2). Several genera significantly distinguish non-CKD controls from overall CKD patients. Among them, a greater AUC in discriminating CKD from the controls was achieved for* Paraprevotella* (AUC, 0.78; 95% CI, 0.7-0.87) and *Pseudobutyrivibrio* spp. (AUC, 0.76; 95% CI, 0.67-0.84) than the use of urine protein/creatinine ratio (AUC, 0.755), and their performance was further replicated in a validation cohort (Table 2). Of note, at the species level, *Collinsella stercoris* exhibited a superb efficacy in discriminating controls from patients with all different levels of CKD severities, with an AUC of 0.83 (95% CI, 0.78-0.94) for distinguishing controls from the early-stage patients (Table 2 and Table 3). Our results reveal promising avenues for prognosis monitoring and early-stage diagnosis of CKD via specific gut microorganisms.

### Gut bacterial genera correlated with serum levels of IS and pCS

**Figure 6:**
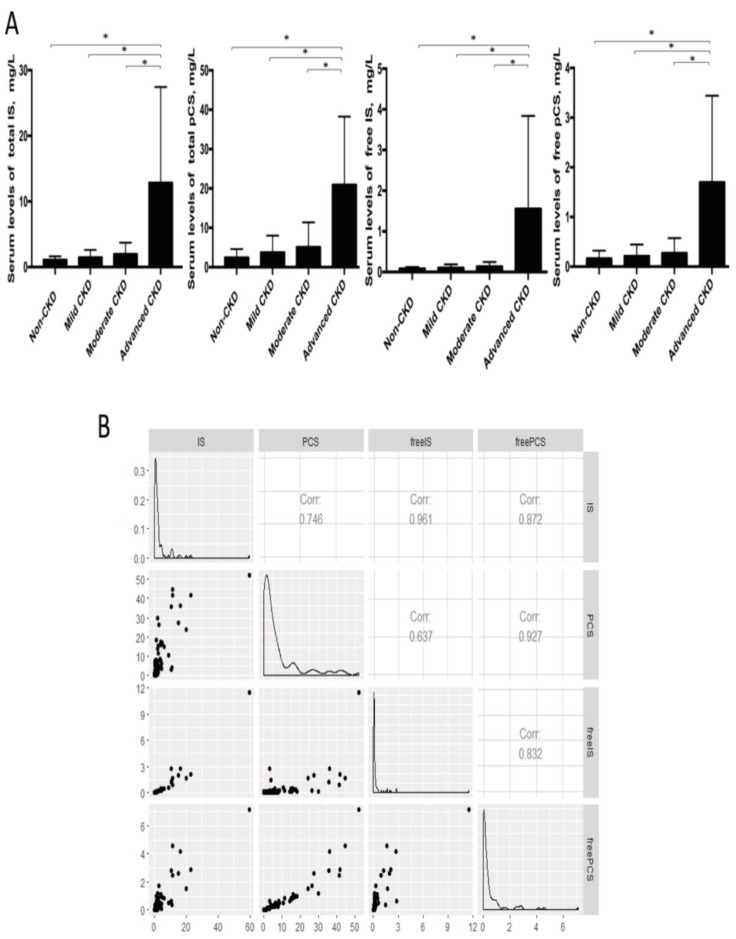
** (A)** Serum levels of uremic toxins at different CKD stages were compared by using Student's t test. * p<0.001. IS, indoxyl sulfate; pCS, p-cresyl sulfate. **(B)** Correlation among free-form and total uremic toxins.

Considering the long-lasting notion that protein fermentation by gut microbiota may generate metabolites with renal toxicity, we further measured the free-form and total (free-form and protein-bound) IS and pCS, two gut-derived uremic solutes, in the serum samples of our cohorts. The levels of free IS and pCS were highly correlated with that of total circulating IS and pCS, respectively (Figure 6). Both IS and pCS levels reflected the degree of renal impairment and peaked at the advanced stage of CKD. To explore the potential interaction between fecal bacterial flora and gut-producing uremic toxins, the relationships of IS and pCS levels with gut microbial profiles at the genus level were assessed. The abundance of 3 CKD stage-correlated genera identified above (*Pseudobutyrivibrio*, *Dialister* and* Escherichia_Shigella*) was also found to be highly correlated with both total and free serum levels of IS. In addition, a significant correlation of *Ruminiclostridium_5* and *Ruminococcaceae_UCG_002* was obtained with the amounts of not only total and free IS but also both forms of pCS. However, the correlation between *Alistipes* and IS (or pCS) was significant only for the total-forms but not for the free-forms. This correlation was similar for *unidentified_Ruminococcaceae* (Table 4).

### Functional prediction of intestinal microbiota at different stages of CKD

**Figure 7:**
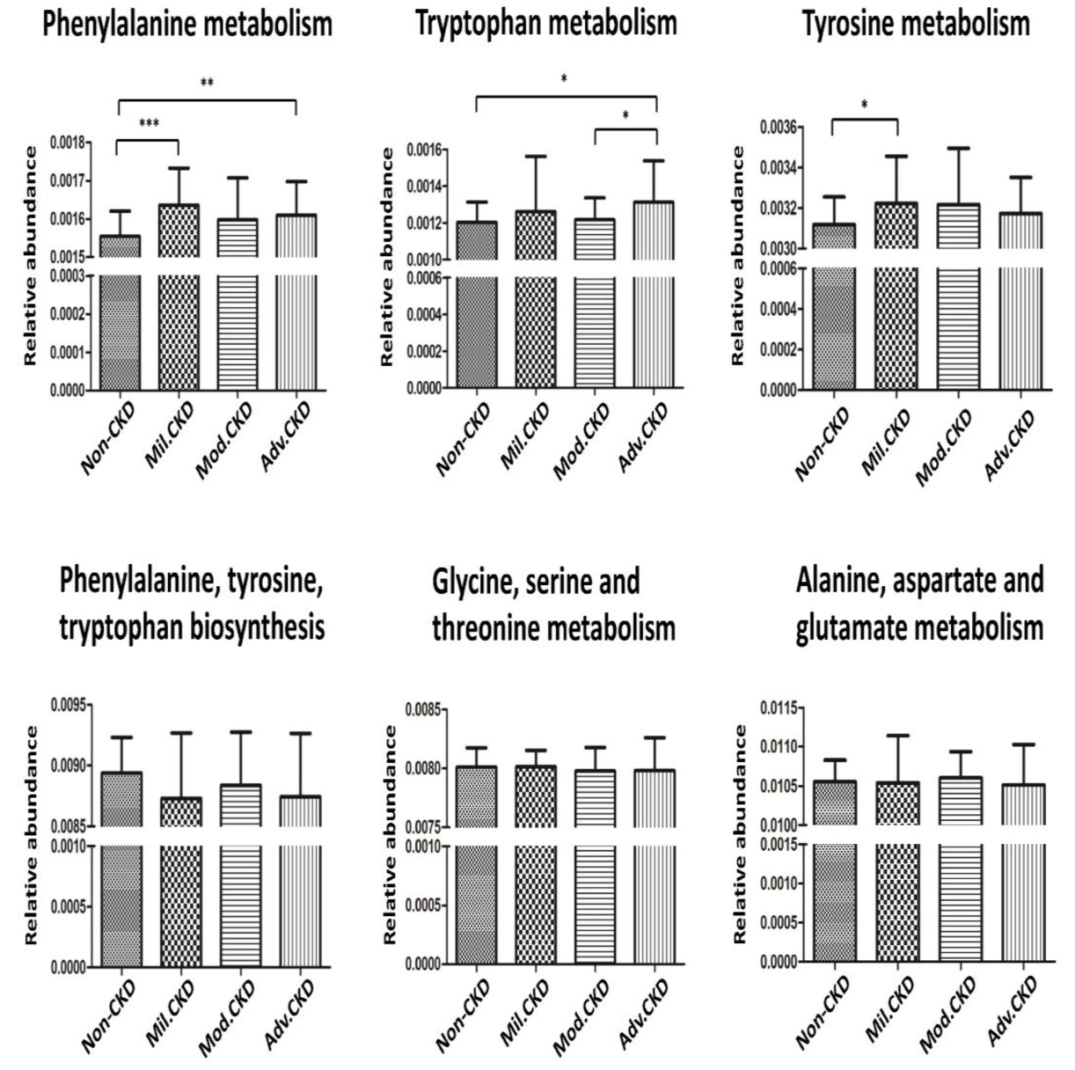
** Prediction of microbial gene functions across different CKD stages.** Pathway enrichment for KEGG metabolism was inferred by PICRUSt. Difference in relative abundance of predicted microbial genes related to the metabolism and biosynthesis of aromatic and other amino acids among non-CKD controls and different stages of CKD was analyzed using Student's t test. *,* p* <0.05; **,* p* <0.01; ***,* p* <0.001.

The cross-domain relationship among the CKD severity, serum levels of uremic toxins, and dysbiosis of gut microbiome noted in the present study may suggest functional interaction. To gain an insight into the functionality of fecal microbiota in CKD etiology, we inferred the functional profile of bacterial communities by PICRUSt 35. We found that in addition to the difference detected in bacterial composition and diversity, microbial genes related to the metabolism of aromatic amino acids (phenylalanine, tyrosine, and tryptophan) were differentially enriched among the control and different CKD stages (Figure 7). Notably, such fluctuation in gut microbial function taking place at the initial stage of disease course was observed for neither the biosynthesis of aromatic amino acids nor the metabolism of other amino acids. Collectively, data shown in the present study indicated that compositional and functional changes in gut microbiome occur since the beginning of disease progression and are associated with the levels of toxic metabolites and disease severity in CKD.

## Discussion

Dysbiosis of gut microbiome, which results in generation of excessive nephrotoxins, may dictate the development and progression of CKD. However, current culture-independent studies of gut microbiome on CKD have been mostly focused on the advanced stage of disease 9-11, 20-22. In the present study, through analyzing the fecal samples from subjects with normal renal function and CKD patients with different disease severities, we identified intestinal bacterial biomarkers that are highly discriminatory since the early stage and mirror the disease severity of CKD. The clinical validity of these CKD-associated microbes was examined and further replicated in an independent cohort. In addition to the disease severity, bacterial genera highly correlated with serum levels of two microbiota-derived nephrotoxins, pCS and IS, were identified, and predicting the functional capabilities of gut microbiome revealed that microbial genes related to the metabolism of aromatic amino acids were differentially enriched among the control and different CKD stages. Overall, our findings demonstrate a link of gut-metabolite-kidney axis to the pathogenesis of renal impairment and highlight specific gut microorganisms as useful biomarker for early diagnosis and prognosis monitoring of CKD.

Short-chain fatty acids (SCFAs), produced by gut microbiota-mediated fermentation 41, have been shown to be nephroprotective 42, 43. In 5 core CKD-associated genera identified to be inversely correlated with the disease course (Figure 4), *Roseburia* and *Pseudobutyrivibrio* are butyrate-producing bacteria 44, while *Dialister* spp. generate propionate 41, unlocking potential avenues for CKD management via replenishment of SCFAs by supplementation of selected prebiotics or probiotics. Of note, another significantly and inversely correlated genus, *Lachnospiraceae ND3007 group*, was associated with a decrease in blood glucose levels in diabetic rats 45. This finding, together with ours, indicates that these intestinal microbes may be involved in production of nephroprotective metabolites or depletion of nephrotoxic solutes, thereby being potentially therapeutic candidates for the intervention of CKD or its comorbidities.

In addition to bacterial biomarkers related to CKD severities, we also identified bacterial genera highly associated with serum levels of microbiota-derived nephrotoxins, IS and pCS. Except for two SCFA-producing genera (*Pseudobutyrivibrio* and *Dialister*) that were inversely correlated with the amounts of circulating IS and may serve as IS-suppressing bacteria, we showed that *Escherichia_Shigella* spp, previously found to be dominated in the urine of CKD patients 46 and feces of ESRD patients 11, were not only consistently enriched in the advanced stage and PD patients but also highly associated with the levels of IS in CKD patients. It is well documented that *Escherichia coli* can convert tryptophan into indole 47. We demonstrated that *Escherichia coli* was identified as the biomarker for the advanced CKD and yielded an AUC of 0.98 (95% CI=0.94-1) in discriminating PD patients from controls in our validation cohort. Our results suggested that *Escherichia_Shigella* spp. overrepresented in the advanced CKD were functionally implicated in the process of renal impairment via excessive IS production. Although numerous bacteria involved in producing IS or pCS have been identified in *in vitro* and animal experiments 48, studies using clinical specimens of patients with renal impairment to search for intestinal microbes associated with IS or pCS are scarce. As a small-scale study of ESRD failed to reach conclusive results 10, another investigation using the TwinsUK cohort with early renal decline found that microbes belonging to the family of *Ruminococcaceae* were associated with IS and pCS 12. Consistently, most of pCS-associated and IS-associated genera (Table 4) identified in the present study pertain to this family of anaerobes. Recently, genetic manipulation of the gut *Bacteroides* by the deletion of the gene encoding tryptophanase can eliminate the production of indole and modulate IS levels in gnotobiotic mice 49. Despite these appealing results, further clinical trials should be warranted to demonstrate reduction of IS and pCS through manipulation of these intestinal microbes.

These potentially pathogenic associations, further supported by our functional prediction that bacterial genes related to the metabolism of aromatic amino acids were differentially enriched across different CKD stages, highlight the solid contribution of gut-metabolite-kidney axis on the pathogenesis of CKD.

Furthermore, clinical validity of gut bacterial biomarkers identified was assessed and verified by using two independent cohorts. Mostly, the higher the severity of renal impairment is, the better performance these CKD stage-associated biomarkers can achieve (Table 2). *Paraprevotella* spp., belonging to the family of Prevotellaceae*, were decreased with the progression of CKD and showed a better* discernibility in discriminating the controls from not only overall CKD but also from early-stage patients than did the use of urine protein-creatinine ratio. It is reported that a decrease of Prevotellaceae* family was observed in rats with uremia 9. Strikingly, we demonstrated that Collinsella stercoris* yielded the best performance of differentiating the controls from all different levels of CKD severities. *Collinsella stercoris* has been shown to be associated with dyslipidemia 50, a common complication among CKD patients. However, no difference in triglycerides, cholesterol, and LDL-cholesterol was detected between the controls and CKD groups in the present study (Table 1). The genomic prediction of functionality of this specific microbe indicated involvement on the oxidation-reduction process of cellular proteins. However, the underlying pathophysiological mechanisms of *Collinsella stercoris* for development of renal toxicity deserve further investigation. Here, we reported a high discriminatory gut microbe species as gut biomarker for early diagnosis and potentially prognosis of renal diseases.

Several limitations should be addressed in this study, including unique ethnic group, differences of eating behaviors and diet composition, inference of functional capacities of bacterial communities based on 16S rRNA gene sequencing. However, the inclusion of patients with wide range of renal abnormalities, recoding of daily protein intakes, use of two independent cohorts to develop and validate the gut biomarkers and the matching strategies for non-CKD controls to minimize confounding effects from baseline characteristics, may all strengthen the conjecture of our supposition. We found a graded change on gut microbiota composition (Figure 1) and on the correlation of levels of core-CKD microbiota (Figure 4) across different disease stage in the discovery cohort. In addition, we demonstrated that the levels of IS and pCS were highest among advanced CKD patients, and, the differences of prediction of microbial genes functions on the metabolism of phenylalanine and tryptophan were significant between non-CKD and advanced CKD patients. To further explore the impact of our candidate microbes responsible for renal disease severity, we decided to include the gravest end-stage renal disease patient to validate our finding. Since PD patients received home-based 24h continuous renal replacement therapy using the own peritoneum as dialyzer membranes, they may have less problem with biocompatibility, dietary and fluid control and better residual renal function than hemodialysis patients. The vegetable and fruit intake were less limited in PD than hemodialysis ones, leading to lower occurrence of constipation in the former group of patients 22. Crespo-Salgado et al. found a significant decrease of α-diversity and an increase of glucose fermentable bacteria, such as the Enterobacteriaceae in PD patients, because of increased intestinal absorption of glucose from the PD dialysate. We found that the loss of α-diversity was present among different CKD stage patients, especially the moderate stage patients (Figure 2E). In spite of higher dialysate glucose absorption in PD patients, the increase of Enterobacteriaceae was also present in our CKD patients (data not shown). Hence, the differences of α-diversity and proportion of Enterobacteriaceae were less likely to have impact on the results of our study, by using PD patients as validation cohort. AST-120 is an oral carbonaceous nanoparticle able to bind IS and pCS, thereby allowing the clearance of these toxins in the feces. Previously, the use of AST-120 was associated with change of microbial composition in CKD rats 51; nevertheless, the renal protective effect associated with its administration remains inconclusive 52, 53. It is unclear if use of AST-120 can reverse the gut microbial change attributed to different CKD severity in humans. For this reason, we enrolled a small sample of patients with AST-120 treatment for assessment of bacterial composition and diversity (Figure 3). However, these 8 patients were excluded for further analysis in searching of core CKD-associated microbiota because of possible influence of AST-120 (rather than the CKD stage *per se*) and limited sample size on the results of o study. Overall, we showed that intestinal microbiota is compositionally and functionally associated with the disease severity and circulating nephrotoxin levels in CKD patients. Our study implicates specific gut microbes as a potential biomarker for early diagnosis and prognosis monitoring in this global health burden, and may serve as candidate therapeutic targets for gut-metabolite-kidney intervention. Further clinical trials should be needed to evaluate the effects of manipulation of these specific microbes in the reduction of levels of nephrotoxins and in the improvement of renal outcome of CKD patients.

## Figures and Tables

**Table 1:** Baseline characteristics of study population (n=130)

	Non-CKD (n=30)	Mild CKD (n=31)	Moderate CKD (n=30)	Advanced CKD (n=31)	AST-120 (n=8)
Age, years	61.6 ± 8.7	62.4 ± 4.1	63.6 ± 6.1	66.2 ± 7.4	67 ± 10.1
Male gender, n (%)	12 (40%)	14 (45.2%)	18 (60%)	15 (48.4%)	2 (25%)
Diabetes, n (%)	19 (63.3%)	16 (51.6%)	15 (50%)	17 (54.8%)	3 (37.5%)
Hypertension, n (%)	27 (87.1%)	23 (76.7%)	26 (86.7%)	30 (96.8%)	6 (75%)
Diastolic pressure, mm Hg	74.1 ± 9.5	75.4 ± 12.1	75 ± 8.4	75.3 ± 12.3	71.6 ± 5.6
Systolic pressure, mm Hg	130.1 ± 18.4	132.3 ± 17.2	127.3 ± 13.9	137.9 ± 14.3	139.8 ± 16.2
Body mass index, Kg/m2	25.5 ± 3.4	27.5 ± 3.6	26.5 ± 4.2	25.9 ± 4.3	25.9 ± 4.3
Waist, cm	85.3 ± 8.8	92.7 ± 11.1	88.6 ± 7.5	88.6 ± 10.7	91.5 ± 8.02
Blood urea nitrogen, mg/dL	13.4 ± 3.9	16.5 ± 4.8	19.6 ± 5.9	61 ± 26.1*	69.3 ± 39.4*
Serum creatinine, mg/dL	0.7 ± 0.2	1.0 ± 0.2	1.4 ± 0.5	4.4 ± 2.3*	6.1 ± 2.9*
Estimated GFR, mL/min/m2	112.4 ± 54.4	71.4 ± 22.1*	49.9 ± 10.2*	16.2 ± 10*	14.1 ± 18.6*
UPCR, g/g#	87.6 (87.2)	136.6 (347.8)	104.3 (171.9)	1596.1 (2305.8)	1348.6 (1060.5)
Hemoglobin, g/dL	13.3 ± 0.9	13.9 ± 1.3	12.9 ± 1.3	10.0 ± 1.9*	9.6 ± 1.6*
Serum albumin, mg/dL	4.6 ± 0.3	4.6 ± 0.3	4.5 ± 0.3	4.1 ± 0.6*	4.2 ± 0.4*
Total cholesterol, mg/dL	191.42 ± 30.77	186.52 ± 25.32	171.5 ± 29.26	193.66 ± 47.84	152.56 ± 46.35
LDL-cholesterol, mg/dL	111.46 ± 30.61	107.55 ± 22.58	95.35 ± 25.33	109.05 ± 35.16	86.06 ± 40.58
Triglyceride, mg/dL	159.39 ± 99.92	129.61 ± 65.29	141.8 ± 83.11	182.25 ± 153.55	115 ± 81.04
hs-CRP, mg/L#	1.23 (1.45)	1.37 (2.15)	1.19 (1.45)	2.23(7.88)	1.07 (1.49)
Estimated protein intake, g/day	77.9 ± 28.2	70.6 ± 22.6	58.6 ± 21	58.6 ± 21.1	57.5 ± 20.6

Data are expressed in mean (SD) or median (interquartile range)#. Abbreviation: CKD, chronic kidney disease; GFR, filtration glomerular rate; hs-CRP, high sensitive C reactive protein; UPCR, urine protein-creatinine ratio. * p < 0.005 vs normal. Estimated protein intake (g/day) = 6.25 × [Urine urea nitrogen (g/day) + 30 mg/kg/day × Weight (kg)].

**Table 2:** Clinical validity for potential biomarkers in discriminating the controls from CKD patients with different severities.

Biomarker		Validation cohort					
		Non-CKD (30) vs. mild CKD (31)		Non-CKD (30) vs. overall CKD (92)		Non-CKD (22) vs. PD (76)	
		AUC (95% CI)	P value/Pc value	AUC (95% CI)	P value/Pc value	AUC (95% CI)	P value/Pc value
**Bacterial genus**							
Tyzzerella 3↓		0.73 (0.60-0.86)	0.002/0.2	0.86 (0.79-0.92)	<0.001/**<0.001**	n.d.	n.d.
Paraprevotella↓		0.76 (0.64-0.88)	<0.001/**0.05**	0.78 (0.70-0.87)	<0.001/**<0.001**	0.76 (0.62-0.89)	<0.001/**0.02**
Lachnospiraceae ND3007 group↓		0.75 (0.62-0.87)	<0.001/0.09	0.78 (0.70-0.87)	<0.001/**<0.001**	0.64 (0.50-0.77)	0.04/4.1
Eubacterium ruminantium group↓		0.66 (0.52-0.81)	0.03/2.842	0.77 (0.69-0.86)	<0.001/**<0.001**	n.d.	n.d.
Pseudobutyrivibrio↓		0.7 (0.57-0.84)	0.006/0.6	0.76 (0.67-0.84)	<0.001/**0.002**	0.72 (0.62-0.82)	<0.001/**0.01**
Lactobacillus↑		0.66 (0.51-0.80)	0.04/3.528	0.74 (0.62-0.86)	<0.001/**0.009**	0.89 (0.81-0.97)	<0.001/**<0.001**
Dialister↓		0.68 (0.55-0.82)	0.01/1.372	0.73 (0.63-0.83)	<0.001/**0.01**	0.75 (0.64-0.87)	<0.001/**0.02**
**Bacterial species**							
Collinsella stercoris↑		0.83 (0.72-0.93)	<0.001/**<0.001**	0.86 (0.78-0.94)	<0.001/**<0.001**	0.94 (0.88-1.00)	<0.001/**<0.001**
Bacteroides eggerthii↓		0.79 (0.67-0.91)	<0.001/**0.007**	0.81 (0.72-0.91)	<0.001/**<0.001**	0.59 (0.45-0.73)	0.2/4.6
Streptococcus anginosus↑		0.64 (0.50-0.78)	0.06/4.96	0.76 (0.67-0.86)	<0.001/**0.001**	n.d.	n.d.
Bacteroides clarus↓		0.73 (0.61-0.86)	0.002/0.1	0.75 (0.66-0.84)	<0.001/**0.003**	n.d.	n.d.
Bacteroides plebeius↓		0.58 (0.44-0.73)	0.2/20.8	0.74 (0.65-0.83)	<0.001/**0.007**	0.81 (0.71-0.91)	<0.001/**<0.001**
Parabacteroides goldsteinii↓		0.74 (0.62-0.87)	0.001/0.09	0.74 (0.64-0.83)	<0.001/**0.008**	0.61 (0.47-0.74)	0.1/2.0
Bacteroides massiliensis↓		0.66 (0.52-0.80)	0.03/2.4	0.73 (0.65-0.82)	<0.001/**0.009**	n.d.	n.d.
Phascolarctobacterium faecium↑		0.73 (0.60-0.85)	0.002/0.2	0.73 (0.62-0.84)	<0.001/**0.01**	n.d.	n.d.
Bacteroides stercoris↓		0.73 (0.60-0.86)	0.002/0.1	0.72 (0.63-0.81)	<0.001/**0.02**	n.d.	n.d.
Lactobacillus salivarius↑		0.66 (0.5-0.80)	0.03/2.8	0.72 (0.60-0.84)	<0.001/**0.02**	0.74 (0.68-0.80)	<0.001/**<0.001**
Ruminococcus callidus↓		0.57 (0.42-0.72)	0.3/29.6	0.72 (0.62-0.81)	<0.001/**0.03**	n.d.	n.d.
Blautia hydrogenotrophica↓		0.68 (0.54-0.82)	0.01/1.2	0.71 (0.61-0.81)	<0.001/**0.04**	n.d.	n.d.
**Conventional biomarker**							
Urine protein-creatinine ratio		0.675 (0.54-0.81)	0.02	0.755 (0.67-0.84)	<0.001		

CKD, chronic kidney disease; PD, peritoneal dialysis; AUC, the total area under the ROC curve. Corrected P (Pc) values were adjusted by using Bonferroni's correction (Discovery: n=80, for species; n=98, for genus; Replication: n=23, for species; n=87, for genus). ↑ and ↓ indicate an ascending and descending trend of bacterial abundance with the disease progression, respectively. n.d., not determined.

**Table 3:** Total area under the ROC curve for potential biomarkers in discriminating the controls from patients with moderate and advanced CKD.

Biomarker	Non-CKD (30) vs. moderate CKD (30)			Non-CKD (30) vs. advanced CKD (31)	
	AUC (95% CI)	P value/Pc value		AUC (95% CI)	P value/Pc value
**Bacterial genus**					
Tyzzerella 3	0.93 (0.85-1.00)	1.5E-08/**1.47E-06**		0.92 (0.83-1.00)	1.9E-08/**1.862E-06**
Paraprevotella	0.76 (0.62-0.89)	0.00062/0.06076		0.83 (0.73-0.93)	1.2E-05/**0.001176**
Lachnospiraceae ND3007 group	0.72 (0.59-0.86)	0.0029/0.2842		0.87 (0.79-0.96)	5.7E-07/**5.586E-05**
Eubacterium ruminantium group	0.86 (0.75-0.96)	1.9E-06/**0.0001862**		0.8 (0.68-0.92)	5.3E-05/**0.005194**
Pseudobutyrivibrio	0.72 (0.58-0.85)	0.0037/0.3626		0.85 (0.75-0.95)	6.9E-07/**6.762E-05**
Lactobacillus	0.82 (0.70-0.93)	2.6E-05/**0.002548**		0.74 (0.61-0.88)	0.0011/0.1078
Dialister	0.65 (0.51-0.80)	0.041/4.018		0.84 (0.75-0.94)	3.9E-06/**0.0003822**
**Bacterial species**					
Collinsella stercoris	0.9 (0.82-0.98)	1.0E-07/**8.0E-06**		0.86 (0.76-0.96)	1.5E-06/**0.00012**
Bacteroides eggerthii	0.88 (0.78-0.97)	4.9E-07/**3.92E-05**		0.77 (0.65-0.90)	0.00024/**0.0192**
Streptococcus anginosus	0.85 (0.75-0.95)	2.6E-06/**0.000208**		0.8 (0.69-0.92)	5.1E-05/**0.00408**
Bacteroides clarus	0.85 (0.75-0.95)	2.8E-06/**0.00232**		0.66 (0.53-0.80)	0.028/2.24
Bacteroides plebeius	0.82 (0.70-0.93)	2.9E-05/**0.00232**		0.82 (0.71-0.93)	1.9E-05/**0.00152**
Parabacteroides goldsteinii	0.79 (0.67-0.91)	0.00011/**0.0088**		0.68 (0.54-0.82)	0.017/1.36
Bacteroides massiliensis	0.74 (0.60-0.88)	0.0013/0.104		0.8 (0.69-0.92)	5.3E-05/**0.00424**
Phascolarctobacterium faecium	0.78 (0.65-0.90)	0.00026/**0.0208**		0.69 (0.55-0.83)	0.011/0.88
Bacteroides stercoris	0.78 (0.64-0.91)	0.00026/**0.0208**		0.67 (0.52-0.81)	0.025/2
Lactobacillus salivarius	0.8 (0.67-0.93)	6.7E-05/**0.00536**		0.7 (0.56-0.84)	0.0085/0.68
Ruminococcus callidus	0.79 (0.66-0.91)	1.4E-04/**0.0112**		0.79 (0.68-0.91)	0.00008/**0.0064**
Blautia hydrogenotrophica	0.9 (0.82-0.98)	1.1E-07/**8.8E-06**		0.56 (0.41-0.71)	0.44/35.2
**Conventional biomarker**					
Urine protein-creatinine ratio	0.603 (0.46-0.75)	0.169		0.981 (0.95-1.00)	<0.001

CKD, chronic kidney disease; AUC, the total area under the ROC curve. Corrected P (Pc) values were adjusted by using Bonferroni's correction (n=80, for species; n=98, for genus).

**Table 4:** Gut bacterial genera correlated with serum levels of indoxyl sulfate or p-cresyl sulfate in discovery cohort

Indoxyl sulfate	Total-form				Free-form		
Genus	R	P-value	Adjusted P		R	P-value	Adjusted P
Ruminiclostridium_5	0.408250	0.000004	0.000245		0.364685	0.000077	0.004241
Dialister	-0.390723	0.000012	0.000671		-0.410164	0.000007	0.000389
Escherichia_Shigella	0.361672	0.000057	0.003150		0.413255	0.000006	0.000327
Alistipes	0.359485	0.000064	0.003510		0.276417	0.003176	0.174654
unidentified_Ruminococcaceae	0.342554	0.000146	0.008050		0.297251	0.001459	0.080226
Pseudobutyrivibrio	-0.338714	0.000176	0.009660		-0.410466	0.000007	0.000383
Desulfovibrio	0.311456	0.000597	0.032800		0.278645	0.002930	0.161158
Ruminococcaceae_UCG_002	0.301976	0.000890	0.048900		0.257879	0.006049	0.332694
Anaerostipes	-0.300192	0.000958	0.052700		-0.422023	0.000004	0.000197
Ruminiclostridium	-0.291262	0.001375	0.075600		-0.403412	0.000010	0.000568
Ruminococcaceae_UCG_004	0.285329	0.001737	0.095500		0.234490	0.012826	0.705415
Ruminococcaceae_UCG_005	0.278029	0.002301	0.127000		0.235933	0.012269	0.674768
**p-Cresyl sulfate**	**Total form**				**Free form**		
**Genus**	**R**	**P-value**	**Adjusted P**		**R**	**P-value**	**Adjusted P**
Ruminiclostridium_5	0.447455	<0.0001	0.000021		0.468585	0.000001	0.000033
Alistipes	0.445359	<0.0001	0.000024		0.400767	0.000027	0.001501
Ruminococcaceae_UCG_002	0.383635	0.000018	0.000990		0.361680	0.000174	0.009581
Ruminococcaceae_UCG_005	0.379890	0.000022	0.001220		0.372297	0.000108	0.005926
unidentified_Ruminococcaceae	0.371433	0.000035	0.001900		0.284746	0.003555	0.195513
Clostridium_sensu_stricto_1	0.337451	0.000186	0.010200		0.349603	0.000295	0.016222
Ruminococcaceae_NK4A214_group	0.320452	0.000404	0.022200		0.362512	0.000168	0.009233
Ruminococcaceae_UCG_004	0.318145	0.000447	0.024600		0.322497	0.000893	0.049107
Christensenellaceae_R_7_group	0.290221	0.001433	0.078800		0.320379	0.000970	0.053323
Eubacterium_coprostanoligenes_gr	0.289604	0.001468	0.080800		0.254246	0.009554	0.525487
Dialister	-0.281914	0.001983	0.109000		-0.309122	0.001487	0.081799
Escherichia_Shigella	0.278791	0.002235	0.123000		0.338280	0.000474	0.026084

Bacterial genera present at or above 0.1% of the total were analyzed by the Spearman's rank correlation test. Corrected P values were adjusted by using Bonferroni's correction (n=55).

## Abbreviations

AUC: area under curve
CKD: chronic kidney disease
eGFR: estimated glomerular filtration rate
ESRD: end-stage renal disease
IS: indoxyl sulfate
LDA: linear discriminant analysis
LEfSe: linear discriminant analysis of effect size
NMDS: non-metric dimensional scaling
OTUs: operational taxonomic units
PCoA: principal coordinate analysis
PCR: polymerase chain reaction
pCS: p-cresyl sulfate
PD: peritoneal dialysis
PICRUSt: phylogenetic reconstruction of unobserved states
ROC: receiver operating characteristic
SCFAs: short-chain fatty acids
UPLC-MS/MS: ultraperformance liquid chromatography-tandem mass spectrometry
V4: variable region 4
WGCNA: weighted correlation network analysis
